# Validation of two short versions of the Zarit Burden Interview in the palliative care setting: a questionnaire to assess the burden of informal caregivers

**DOI:** 10.1007/s00520-019-05288-w

**Published:** 2020-02-15

**Authors:** Martina B. Kühnel, Christina Ramsenthaler, Claudia Bausewein, Martin Fegg, Farina Hodiamont

**Affiliations:** Department of Palliative Medicine, Munich University Hospital, LMU Munich, Munich, Germany

**Keywords:** Caregivers, Caregiver burden, Palliative care, Validation studies, Zarit burden interview, Psychometrics

## Abstract

**Purpose:**

Several validated outcome measures, among them the Zarit Burden Interview (ZBI), are valid for measuring caregiver burden in advanced cancer and dementia. However, they have not been validated for a wider palliative care (PC) setting with non-cancer disease. The purpose was to validate ZBI-1 (ultra-short version and proxy rating) and ZBI-7 short versions for PC.

**Methods:**

In a prospective, cross-sectional study with informal caregivers of patients in inpatient (PC unit, hospital palliative support team) and outpatient (home care team) PC settings of a large university hospital, content validity and acceptability of the ZBI and its structural validity (via confirmatory factor analysis (CFA) and Rasch analysis) were tested. Reliability assessment used internal consistency and inter-rater reliability and construct validity used known-group comparisons and a priori hypotheses on correlations with Brief Symptom Inventory, Short Form-12, and Distress Thermometer.

**Results:**

Eighty-four participants (63.1% women; mean age 59.8, SD 14.4) were included. Structural validity assessment confirmed the unidimensional structure of ZBI-7 both in CFA and Rasch analysis. The item on overall burden was the best item for the ultra-short version ZBI-1. Higher burden was recorded for women and those with poorer physical health. Internal consistency was good (Cronbach’s *α* = 0.83). Inter-rater reliability was moderate as proxy ratings estimated caregivers’ burden higher than self-ratings (average measures *ICC =* 0.51; *CI =* 0.23–.69; *p =* 0.001).

**Conclusion:**

The ZBI-7 is a valid instrument for measuring caregiver burden in PC. The ultra-short ZBI-1 can be used as a quick and proxy assessment, with the caveat of overestimating burden.

**Electronic supplementary material:**

The online version of this article (10.1007/s00520-019-05288-w) contains supplementary material, which is available to authorized users.

## Introduction

According to the WHO definition, palliative care (PC) addresses the needs of patients and offers a support system to help the family cope during the patients’ illness and in bereavement [1]. Not only family members but also friends or neighbours can be involved in taking care of a patient, and as long as their support is not financially rewarded, they can be defined as informal caregivers [2].

Informal caregivers can become “patients” themselves, as their psychological morbidity is substantially higher compared with the general population [3]. There is a close relationship between the patient’s perceived burden and that of the caregiver [4, 5], often leading to higher caregiver burden in the later stages of the patient’s illness and a corresponding increase in need for physical and emotional support for caregivers [6, 7].

There is a growing number of intervention programmes [8, 9] aiming at caregiver outcomes, such as reducing caregivers’ burden, improving caregivers’ coping, or their quality of life. However, quantifying the impact of interventions is impossible without validated outcome measures for caregivers. A systematic review by Michels et al. showed that the majority of studies measuring informal caregiver outcomes in PC use carer-specific measures, primarily measures of caregiver burden [10]. According to Michels et al. the Zarit Burden Interview (ZBI) [11] is one of the two most frequently used measures of burden [10], the other one being the caregiver reaction assessment (CRA) [12]. The ZBI, originally comprising 22 items [11, 13, 14], has several short forms including between four and twelve items, and the overall burden is assessed by the total score of all items, with a higher score representing greater caregiver burden [15–17]. Higginson et al. validated ZBI short versions in advanced conditions with caregivers of patients with advanced cancer, dementia, and acquired brain injury (ABI) [18]. The authors recommended using ZBI-6 and ZBI-7 (ZBI-6 plus ZBI-1) in the PC setting as they showed good validity, internal consistency, and discriminatory performance. Additionally, it was reported that the ZBI-1 might be suitable for screening [18].

However, although the ZBI is well-known and used, a formal validation of ZBI short versions in the PC setting using psychometric testing and Rasch analysis, and complementing the results Higginson et al. [18], is still lacking. Furthermore, the German ZBI 22-item version was validated by Braun et al. [19] for female caregivers of dementia patients, but not validated in a German PC setting yet.

The palliative care context differs from dementia due to often rapidly progressing diseases, and caregiving at the end of life causes the greatest caregiver burden. [20]

Therefore, the aim of this study was (1) to test the ZBI-7, the ZBI-6, and the ZBI-1 short versions for content validity, structural validity, construct validity, and reliability in the PC setting; (2) to confirm findings using Rasch analysis; (3) to evaluate the suitability of ZBI-1 as a proxy assessment for staff members; and (4) to evaluate the suitability of ZBI-1 item as an ultra-short instrument for quick assessment based on validity, reliability, and Rasch analysis.

## Methods

### Design

This is a prospective, cross-sectional validation study. Psychometric properties are reported according to the Consensus-based Standards for the selection of health status Measurement Instruments (COSMIN) guidelines [21, 22] and the quality criteria for measurement properties of health status questionnaires by Terwee et al. [23]. The Ethics Committee of the Ludwig-Maximilians University Munich approved the study (REC-No 772–16).

### Setting and population

The study was conducted in the Department for Palliative Medicine at Munich University Hospital. Informal caregivers of patients treated by the hospital support team, and the home care team were consecutively recruited.

In the inpatient PC unit, questionnaires were included in the pre-intervention assessment of a randomized controlled trial evaluating an intervention for informal caregivers (Clinical.Trials.gov registration NCT02325167). The combination of the two studies was approved by the university’s ethics committee.

Inclusion criteria were being an informal caregiver of a palliative patient, a minimum age of 18 years, proficiency in written and spoken German, and the ability to give written informed consent. Caregivers with poor general condition, caregivers of patients who had been admitted to PC the same day, or who were imminently dying were excluded. Eligibility for inclusion was assessed by a staff member.

All participating caregivers and patients provided written informed consent. Consent of a legal guardian was sought for those patients unable to give consent.

### Data collection

Data were collected between February 2017 and February 2018 using self-assessed questionnaires. Demographic data included age, sex, ethnicity, religion, highest academic qualification, profession, and marital status. Information on type of relation to the patient, role in caring for the patient, and the living status was collected for caregivers. Patient data were collected from medical notes and included age, date of PC admission, symptom burden at day of admission (via routinely collected Integrated PC Outcome Scale [24]), diagnosis, and date of discharge or death.

A member of the attending PC team assessed caregiver burden as a proxy using the ZBI-1 for inter-rater agreement. Staff members were asked for written informed consent and the following demographic data: age, sex, profession, work setting, and years of experience in PC.

Caregivers, who appeared highly burdened personally or in the assessments, were offered additional supportive talks by the multidisciplinary team.

### Measurement instruments

Zarit Burden Interview-7 (ZBI-7): 7-item version of the original 22-item version measuring caregivers’ physical and psychosocial burden on a five-point Likert scale [13, 18]. From the German translation by Braun et al. [19], we chose the seven items (see Table 1) recommended for use in PC by Higginson et al. [18]

Brief Symptom Inventory (BSI): measuring psychological distress and psychiatric disorders with 53 items on a five-point rating scale [25].

Distress thermometer: one-item measure with a 0–10 scale ranging from “No distress” to “Extreme distress” [26].

Short form 12:12-item version of the Short Form Health Survey measuring subjective health status on three- and five-point Likert scales [27].

**Table 1:** Short versions, item wording, and distribution of responses (*n* = 84)

Short versions				Responses (%)					
ZBI 7	ZBI 6	ZBI 1	Items	0	1	2	3	4	Miss
Y	Y		1. Do you feel you do not have enough time for yourself?	*17.9*	21.4	41.7	14.3	4.8	0
Y	Y		2. Do you feel stressed between caring and meeting other responsibilities?	*19.0*	26.2	28.6	20.2	6.0	0
Y	Y		3. Do you feel your relative affects your relationship with others in a negative way?	*56.0*	21.4	16.7	3.6	1.2	1.2
Y	Y		4. Do you feel strained when are around your relative?	*34.5*	27.4	27.4	9.5	1.2	0
Y	Y		5. Do you feel your health has suffered because of your involvement with your relative?	*32.1*	20.2	35.7	8.3	3.6	0
Y	Y		6. Do you feel you have lost control of your life since your relative’s illness?	*48.8*	17.9	20.2	9.5	2.4	1.2
Y		Y	7. Overall, how burdened do you feel in caring for your relative?	6.0	17.9	27.7	44.6	3.6	1.2

Y: yes

Italic: responses > 15%; indicating floor-effects

Miss: percentage of missing items

Possible responses to item 1–6: 0, never; 1, rarely; 2, sometimes; 3, quite frequently; 4, nearly always

Possible responses to item 7: 0, not at all; 1, a little; 2, moderately; 3, quite a bit; 4, extremely

### Analysis

Descriptive statistics were used to describe sample distribution and distribution of responses. Missing data were imputed using expectation-maximization technique as data was missing completely at random, as indicated by the non-significant chi^2^ statistic in Little’s MCAR test performed in SPSS [28].

#### Sample size

Two sample size calculations were conducted to power the study for detecting moderate reliability scores and to allow the detection of medium differences between known subgroups regarding the extent of burden (*d* = 0.3 at a power of 80% and at a significance level of 95%). Sample size estimates ranged from 64 to 144 participants, with a minimum of 90 participants needed to detect known-group differences.

#### Validity analysis

Content validity and acceptability comprised the analysis of ceiling and floor effects, indicated if more than 15% of responses are in the highest or lowest category [23], as well as assessment of acceptability by analysis of missing items and user comments.

Structural validity: confirmatory factor analysis (CFA) were run to confirm that all items load on one latent factor, excluding the existence of subscales [29]. CFA was run with maximum likelihood estimation as it is robust to minor deviations from normality and accounts for missing data [30, 31]. Evaluation of model fit was based on fit indices and on the chi^2^/df-ratio rather than on chi^2^, as the latter reacts sensitively to sample size [32]. A chi^2^/df-ratio between 2 and 3 was regarded as indicative of acceptable data-model fit [32, 33]. Fit indices of CFI/TLI ≥ 0.90 were regarded acceptable, and root mean square error of approximation (RMSEA) < 0.08 was regarded as showing good fit [31].

Construct validity: we tested a priori hypotheses on scale-to-scale correlations with other measures, assuming that high correlations imply high convergent validity and suggest that the two scales measure similar concepts [34]. BSI, Distress Thermometer, and SF-12 were chosen, as they are well-known and established measurement instruments and, while not explicitly validated for caregivers in palliative care, have all been used in studies on this population [35, 36].

Twelve a priori hypotheses were formulated on ZBI-1, ZBI6, and ZBI-7—each correlating significantly with the BSI subscales depression and Global Severity Index, with the Distress Thermometer and the SF-12 subscale Mental Health Composite. Moderate correlations (0.4–0.7) were assumed, as all measures represent different aspects of burden-related caregiver outcomes. The family-wise alpha error rate was Bonferroni-corrected to a value of 0.05/12 = 0.004.

Construct validity was also determined through known-groups comparisons [34]. Eight hypotheses were formulated. We hypothesised that burden would be higher for female caregivers, due to studies suggesting sex differences [37, 38]. Furthermore, a block of hypotheses referred to (a) the relationship between caregivers and patient. It was hypothesised that burden would be higher for (i) parents or partners as losing a child conflicts with life cycle expectations, and losing a partner is ranked as one of the most stressful life events [39]; (ii) those living with the patient as studies suggest that there are more negative consequences for caregivers when caregiving in-house [40]; (iii) those giving physical care to the patient, and (iv) those who had power of attorney or legal guardianship for the patient as we suspected a relationship to burden, since caregivers are neither trained nurses nor legal guardians.

A second block of hypotheses referred to (b) caregivers who felt physically strained which can impact on caregivers’ distress [38]. It was hypothesised that ZBI outcomes would be higher for (i) those who scored high on the SF-12 Physical Health Composite (via median split); those who due to physical health in the past 4 weeks (SF-12) (ii) had accomplished less or (iii) had been limited in work or activities. Parametric tests were used for all comparisons, complemented by non-parametric tests to account for non-normal distribution (*t*-test and Kruskal-Wallis H-test for hypothesis (a, i); *t*-test and Mann-Whitney-U tests for all other hypotheses). Hypotheses were tested using non-imputed data to avoid misleading results due to imputation.

The first block of known-group comparisons was tested to a Bonferroni-corrected alpha of 0.05/8 = 0.006; and the last block to a corrected alpha of 0.05/3 = 0.017.

#### Reliability analysis

Internal consistency was assessed as an aspect of reliability [41]. Cronbach’s α = 0.7–0.9 indicated internal consistency without item redundancy.

Inter-rater reliability between self-rating of burden and proxy rating by a staff member was examined with the Intraclass Correlation Coefficients (ICC) [21] and a two-way mixed model of the type consistency [42]. ICC < 0.5 indicated poor reliability, ICC of 0.5–0.75 moderate, 0.75–0.90 good, and ICC > 0.90 excellent reliability [42].

#### Rasch analysis

Rasch analysis complemented the validity analyses and tested items for use as an ultra-short version. The Rasch measurement model tests validity of unidimensional measures. It assumes that the response to a ZBI item is determined by the level of burden a person experiences (person fit) and the level of burden that the item represents (item fit). The Partial Credit Model was used which does not require equidistant categories and is suitable for ordinal-level data. ZBI-7 and ZBI-6 were compared with each other, and for ZBI-1 the self-rating data was compared with the one-item proxy rating by staff members.

Best-performing item candidates for the ultra-short version ZBI-1 were determined by item fit residuals (<and> 2.5), a summary mean item and person fit close to 0 (with SD = 1), ordered Likert response scale weightings for individual answer categories for each item, and the overall floor and ceiling effect for item parameters to person parameters. Overall model fit was assessed using the X^2^-test [43, 44].

CFA was run using IBM SPSS Amos 25 [45]. Rasch analysis was conducted using RUMM 2030 [46]. For all other analyses, SPSS version 25 was used [47]. A *p* value of < 0.05 was considered significant.

## Results

### Acceptability

Overall, 123 informal caregivers participated. Acceptability was assessed after 39 participants had completed the questionnaires. In open-response text fields, problems with the German translation of “care” were noted. Two participants commented “I don’t nurse” and “No nursing” and 2.6–7.7% of items were missing. We therefore decided to change the wording of the German translation and employed the revised version on a sample of 84 participants. Percentage of missing items dropped to 0–1.2%, and overall, the revised version showed better characteristics than the first version. All following analyses in this study were conducted with data of the revised German version only (*n* = 84).

### Characteristics of participants

**Fig. 1:**
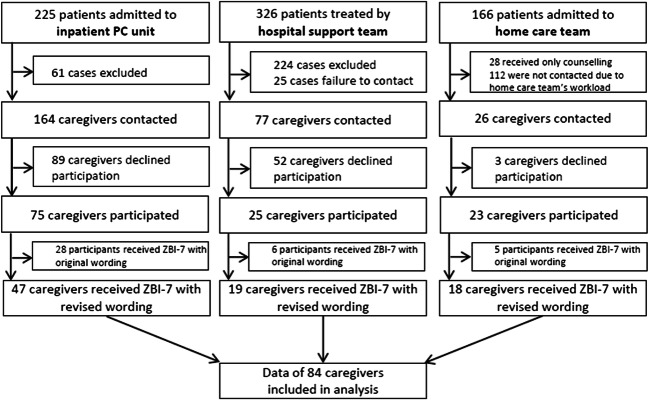
Flow-chart of participants

Data of 84 participants who received the revised ZBI-7 were included in the analyses. Figure 1 shows the participant flow of the three settings. Most participants were female (63.1%; see Table 2); the mean age was 59.8 years (standard deviation (SD) 14.4). Approximately, one third of the participants held a university degree (32.1%), and the majority were married (76.2%). Participants were mostly partners (including wives or husbands) (53.6%) or children (32.1%) of the patients. Cancer was the prevailing diagnosis of the patients (79.8%). For characteristics of participating staff members see electronic supplementary material 1.

**Table 2:** Descriptive characteristics of participants (*n* = 84)

Characteristic	*n*	Percentage
Setting		
Palliative care unit	47	56.0%
Hospital support team	19	22.6%
Home care team	18	21.4%
Age	59.8^a^	14.4^b^
Sex		
Female	53	63.1%
Religion		
Catholic	36	42.9%
None	31	36.9%
Protestant	17	20.2%
Other		
Nationality		
German	82	97.6%
Other	2	2.4%
Education		
University degree	27	32.1%
Upper secondary	12	14.3%
Intermediate secondary	30	35.7%
Lower secondary	13	15.5%
Missing	2	2.4%
Marital status		
Married	64	76.2%
In relationship	13	15.5%
Single	4	4.8%
Divorced/separated	2	2.4%
Widowed	1	1.2%
Relationship with patient (“Patient is my…”)		
Wife/husband	43	51.2%
Partner	2	2.4%
Mother/father	27	32.1%
Sister/brother	7	8.3%
Grandmother/grandfather	1	1.2%
Friend	2	2.4%
Other	2	2.4%
Procuration for patient		
Procuration or legal guardianship	72	85.7%
No procuration or guardianship	12	14.3%
Diagnosis of patient		
Digestive tract cancer	17	20.2%
Genito-urinary cancer	12	14.3%
Brain cancer	8	9.5%
Lung cancer	7	8.3%
Gynaecological cancer	4	4.8%
Breast cancer	3	3.6%
Hemic cancer	3	3.6%
Other cancer	13	15.5%
Neurological disease	8	9.5%
Cardiovascular disease	2	2.4%
Other disease	7	8.3%

^a^ Mean

^b^ SD

### Structural validity

Scores were non-normally distributed for items 3, 6, and 7, with skewness of 1.215 (standard error (SE) = 0.264), 0.842 (SE *=* 0.264), and − 0.582 (SE *=* 0.264), respectively; the latter left-skewed, all others right-skewed. Floor effects were observed for all items except item 7 on overall burden (see Table 1).

The CFA analyses showed a good to moderate fit of a unidimensional model, meaning that all items in the ZBI short versions measure one construct, caregiver burden, only. Fit indices were good (CFI *=* 0.938, TLI *=* 0.907, standardized RMR = 0.0643), and RMSEA was moderate (RMSEA *=* 0.100, 90% confidence interval (CI) = 0.033–0.161). The chi^2^/df-ratio was 1.84, also indicating a good fit to a unidimensional model. Overall, the fit indices and other measures (absence of Heywood cases, meaning negative variances or implausible values for variances and factor loadings) of fit confirm a unidimensional model of caregiver burden and the potential to shorten the ZBI further. All factor loadings were above 0.30, indicating good yet variable ability of individual items in the ZBI to measure the underlying construct of caregiver burden. Factor loadings varied between 0.41 for item 3 and 0.81 for item 7 on overall burden. Item 7 loaded highest onto the latent variable “burden” and showed the highest level of explained variance (see Table 3). ZBI-6 showed lower factor loadings and explained variance, as it lacks the overall item 7. The following results are therefore reported for ZBI-1 and ZBI-7 only.

**Table 3:** Factor loadings of confirmatory factor analyses with EM-imputed data of ZBI-7 and ZBI-6

	ZBI-7		ZBI-6	
ZBI Item	λ	SMC	λ	SMC
1	0.64	0.41	0.66	0.43
2	0.77	0.60	0.79	0.62
3	0.49	0.24	0.51	0.26
4	0.51	0.26	0.48	0.23
5	0.76	0.58	0.75	0.56
6	0.51	0.26	0.50	0.25
7	0.81	0.65	-	-

SMC, squared multiple correlation

λ standardized regression weight (factor loading)

### Convergent validity

Correlations between the ZBI-1 and ZBI-7 scales and individual Zarit items with the Distress Thermometer, the SF-12 Mental Health subscale, the BSI global scale, and BSI depression subscale were analysed. Of the 12 a priori hypotheses nine, 75%, had hypothesised the correct direction of correlations (Bonferroni-corrected alpha level of 0.004; see Table 4).

**Table 4:** A priori hypotheses and results for construct validity using spearman correlation coefficients of the ZBI with SF-12 and BSI (*n* = 84)

Hypothesis	Scales correlated	*rho*	*p*
Moderate^*a*^ convergent validity expected:			
Between ZBI scales and BSI (sub-)scale	*ZBI-1 + BSI Global Severity Index*	*0.41*	*0.000*
	*ZBI-6 + BSI Global Severity Index*	*0.53*	*0.000*
	*ZBI-7 + BSI Global Severity Index*	*0.53*	*0.000*
	ZBI-1 + BSI depression	0.36	0.001
	*ZBI-6 + BSI depression*	*0.45*	*0.000*
	*ZBI-7 + BSI depression*	*0.45*	*0.000*
Between ZBI scales and the Distress Thermometer	*ZBI-1 + Distress Thermometer*	*0.51*	*0.000*
	ZBI-6 + Distress Thermometer	0.35	0.001
	ZBI-7 + Distress Thermometer	0.39	0.000
Between ZBI scales and SF-12 subscale Mental Health Composite	*ZBI-1 + SF12 Mental Health Composite*	*− 0.40*	*0.000*
	*ZBI-6 + SF12 Mental Health Composite*	*− 0.48*	*0.000*
	*ZBI-7 + SF12 Mental Health Composite*	*− 0.49*	*0.000*

^a^Expected correlations: *rho* (0.4)–(0.7)

Italics: correlations that were consistent with hypotheses

### Known-group comparisons

Caregiver burden measured with ZBI-7 was significantly higher for female caregivers The results for the outcome ZBI-1 did not reach statistical significance, based on the Bonferroni-corrected alpha level of 0.006 (ZBI-1 *t* = 2.32, *p* = 0.023; ZBI-7 *t* = 2.96, *p* = 0.004). No hypothesis in block (a) regarding relationship between carers and patient was significant.

In block (b), one of the three hypotheses concerning caregivers who felt physically strained was significant (b ii): Caregiver burden was significantly higher measured with ZBI-7 for those who had indicated on SF-12 that they had accomplished less in the past 4 weeks due to their physical health. The results for the outcome ZBI-1 did not reach statistical significance, based on the Bonferroni-corrected alpha level of 0.0017 (ZBI-1 *t* = 2.01, *p* = 0.048; ZBI-7 *t* = 3.32, *p* = 0.001). Comparisons were also run using non-parametric tests, yielding the same pattern of significant and non-significant results.

### Reliability

Cronbach’s α for the ZBI-7 scale was 0.83 and was reduced with removal of any item. Item 7 on overall burden (ZBI-1) correlated highest with the whole ZBI-7 scale *(r =* 0.73) and if deleted reduced Cronbach’s α most (ZBI-6, Cronbach’s α = 0.78).

ICC was significant for the 1-item ratings by staff members and informal caregivers. Agreement, however, was moderate for average measures (ICC *=* 0.51; CI *=* 0.23–.69; *p =* 0.001). ICCs for the 1-item ratings of staff members and caregivers’ ZBI-7 self-rating were not significant (*p =* 0.211; single measures, ICC *=* 0.09; CI *=* − 0.13–0.31; average measures ICC *=* 0.17; *CI =* − 0.31–0.47).

### Rasch analysis

All three models (ZBI-7, ZBI-6, and ZBI-1) showed good model fit. Mean of ZBI-7 item difficulty was 0.00 (SD = 0.63). Item 3 “affecting relationships” measured the highest levels of burden, while item 7 “overall burden” measured the lowest levels. There was no major deviation from the Rasch model as no item showed residuals of ± 2.5 and all chi^2^ measures were non-significant (Bonferroni-corrected, *p* < 0.001, see electronic supplementary material 2).

The person-item threshold distribution showed a slight mismatch of item and person parameters (see electronic supplementary material 3). Items measured the medium to higher levels of burden. Person parameters (amount of burden as reported by caregivers), however, showed lower to medium values. For ZBI-1, the distribution of scores indicated lower person parameters for caregivers, indicating lower burden, than was observed for staff members’ proxy ratings. Item characteristic curves showed that items 5 “health suffered” and 7 “overall burden” marginally over-discriminated by differentiating well between caregivers with high or low burden. Interval-scale assumption via category probability curves yielded items 2 “meeting responsibilities,” and 4 “feeling strained” as most evenly distributed items. Moreover, item 7 “overall burden,” the designated item of the ZBI-1 ultra-short version, showed comparatively good fit to the Rasch model.

The fit of the self-rated caregiver version (location = 1.172, SE = 0.136, fit residual = − 0.006) was better than the fit of the staff version (location = − 1.172, SE = 0.153, fit residual = 0.715).

## Discussion

Our aim for this study was to close the gap of a formal validation of the ZBI short versions in the PC setting. Additionally, the acceptability of the German ZBI was improved by the change of wording (report in preparation).

Concerning convergent validity, scale-to-scale correlations were significant but moderate, as expected, due to the comparison instruments measuring different aspects of burden-related caregiver outcomes. Two of the eight hypotheses formulated on known groups were significant. As suggested by other studies [37, 38], burden was higher for female caregivers, and for those with poor physical health, which also concurs with other findings [38]. Unlike expected, caregiver burden was not higher for those who were partners or parents, who lived with the patient, physically nursed, or acted as legal guardian.

Our results on reliability for ZBI-7 (Cronbach’s α 0.83) were only minimally higher than in Higginson et al.’s validation (α 0.82). [18]

Analysis of structural validity using CFA and Rasch analysis confirmed the unidimensional structure of the ZBI, allowing for use of the overall score as outcome measure. ZBI-7 showed advantages over ZBI-6 in factor loadings, explained variance, and internal consistency as the additional item 7 on overall burden proved to be the best item and the best choice as the ultra-short version ZBI-1.

Our results concerning ZBI-1 differ from Higginson et al.’s validation study where ZBI-1 for cancer caregivers showed the lowest discriminative ability and the lowest correlation with the 22-item version. Higginson et al. obtained 91% sensitivity and 53% specificity for ZBI-1, meaning that ZBI-1 oversensitively rated most caregivers as burdened [18]. In our study, ZBI-1 showed good fit with the Rasch model, which means that it discriminated very well between high and low burden and only when used as a proxy rating by staff members overestimated caregiver burden.

Using ZBI-1 as a proxy rating, staff members rated caregivers’ level of burden higher than in caregivers’ self-ratings, resulting in mediocre inter-rater reliability. Social desirability could have led to lower self-ratings, as caregivers might have presented themselves as more stable to prevent their ability to care being questioned. A potential consequence of personnel’s higher evaluation of burden could be the provision of support to caregivers who would not have asked for support themselves.

Rasch analysis and analysis of content validity suggested that items were constructed to measure higher levels of burden but caregivers reported lower levels. This may suggest a comparatively poor fit between sample and measure, resulting in false negative ratings of burden. However, participation bias could explain floor effects as participating caregivers possibly felt less burdened than those who decided to decline study participation. Dura and Kiecolt-Glaser reported a similar account of caregiver participation bias [48]. Additionally, caregivers included in this study were recruited from three specialized PC settings, which could have resulted in them being less burdened than caregivers who receive less professional support. Similarly, Higginson et al. reported lower levels of burden for advanced cancer caregivers, who had been recruited solely from specialized support facilities, while caregivers of patients with dementia and ABI showed higher levels of burden and had been recruited from diverse settings [18].

A strength of this study is that it is the first validation study of ZBI short versions that focusses on the PC setting alone. Participants were recruited in all three relevant PC settings. Additionally, this validation study was conducted with methods based on classical test theory and with Rasch analysis, which comprises aspects of item-response theory. Reliability of the ZBI-7 was higher than in previous studies and relative reliability was tested using inter-rater agreement. While the ZBI is well-known and used, our study closes the gap of a formal validation in the PC setting.

Limitations include rather low participant numbers in the home care setting due to low home care team staffing situation and high workload. Therefore, initially only few caregivers had been contacted in this setting, and reasons for exclusion were not recorded consecutively. Inclusion decisions were hence recorded by a member of the study team. Additionally, it must be noted that the recruitment of the biggest part of caregivers was combined with an intervention study, to both preserve resources and spare caregivers, but the approach might have influenced caregivers’ self-ratings. This study provides good validity for ZBI-1 as a proxy rating and potential as an ultra-short instrument, but because of lacking resources further analyses, e.g., of sensitivity or specificity, were not possible. Sample size was slightly smaller than the minimum of 90 participants needed to detect known-group differences, and subgroup comparison was infeasible due to unequal proportion of settings. However, results were obtained by combining methods of classical test theory and Rasch analysis and can therefore be regarded as robust.

In conclusion, this study complements earlier results of Higginson et al. [18]. ZBI-1 and ZBI-7 were shown to be valid in the PC context. ZBI-1 shows promising indication for use as an ultra-short instrument for caregiver burden while ZBI-7 could be used for more comprehensive measurement of caregiver burden, for example, when quantifying the impact of interventions aimed at caregivers in clinical trials and evaluation studies.

## Electronic supplementary material

ESM 1 (DOCX 14 kb)

ESM 2 (DOCX 14 kb)

ESM 3 (DOCX 86 kb)
