# Supplementary material for: Validation of two short versions of the Zarit Burden Interview in the palliative care setting: a questionnaire to assess the burden of informal caregivers
Source: Support Care Cancer. 2020 Feb 15;28(11):5185–93. doi: 10.1007/s00520-019-05288-w (PMC7546983; doi:10.1007/s00520-019-05288-w)
Supplement: Supplementary file 1 — (DOCX 14 kb) [file 520_2019_5288_MOESM1_ESM.docx]

**Table 5** Descriptive Characteristics of Staff Members who rated Caregiver Burden using ZBI-1 (n=22)

| Characteristic | | n (%) or mean (SD) | |
| --- | --- | --- | --- |
| Setting | |  |  |
|  | Palliative care unit | 13 | (59.1%) |
|  | Hospital support team | 4 | (18.2%) |
|  | Home care team | 5 | (22.7%) |
| Age | | 36.5 | (8.83) |
| Sex | |  |  |
|  | Female | 16 | (72.7%) |
| Profession | |  |  |
|  | Nursing staff | 15 | (68.2%) |
|  | Medical staff | 7 | (31.8%) |
| Years of professional experience | | 13.3 | (10.5) |
| Years of experience in palliative care | | 3.3 | (4.6) |
| Number of Zarit-Ratings | | 3.6 | (2.6) |
|  | |  |  |
