# Supplementary material for: Validation of two short versions of the Zarit Burden Interview in the palliative care setting: a questionnaire to assess the burden of informal caregivers
Source: Support Care Cancer. 2020 Feb 15;28(11):5185–93. doi: 10.1007/s00520-019-05288-w (PMC7546983; doi:10.1007/s00520-019-05288-w)
Supplement: Supplementary file 2 — (DOCX 14 kb) [file 520_2019_5288_MOESM2_ESM.docx]

**Table 6** Summary fit statistics of Rasch analysis for scales ZBI-1, ZBI-6 and ZBI-7

| Model | Analysis number | n | Overall model fit | Item fit Mean (SD) | Person fit Mean (SD) | Person Separation Index |
| --- | --- | --- | --- | --- | --- | --- |
| ZBI-7 | 1 | 84 | *X²* = 20.255, df = 14 p = 0.122 | 0.00 (0.63) | -0.91 (1.25) | 0.81 |
| ZBI-7 removal of extremes | 2 | 80 | *X²* = 23.782, df = 14 p = 0,058 | 0.00 (0.61) | -0.87 (1.15) | 0.79 |
| ZBI-6 | 3 | 77 | *X²* = 16.418, df = 12 p = 0.173 | 0.00 (0.43) | -0.84 (0.92) | 0.69 |
| ZBI-1 | 4 | 84 | *X²* = 3.106, df = 4 p = 0.540 | 0.00 (1.66) | 1.23 (1.14) | 0.25 |
