# Supplementary figures and images for: Validation of two short versions of the Zarit Burden Interview in the palliative care setting: a questionnaire to assess the burden of informal caregivers
Source: Support Care Cancer. 2020 Feb 15;28(11):5185–93. doi: 10.1007/s00520-019-05288-w (PMC7546983; doi:10.1007/s00520-019-05288-w)

**
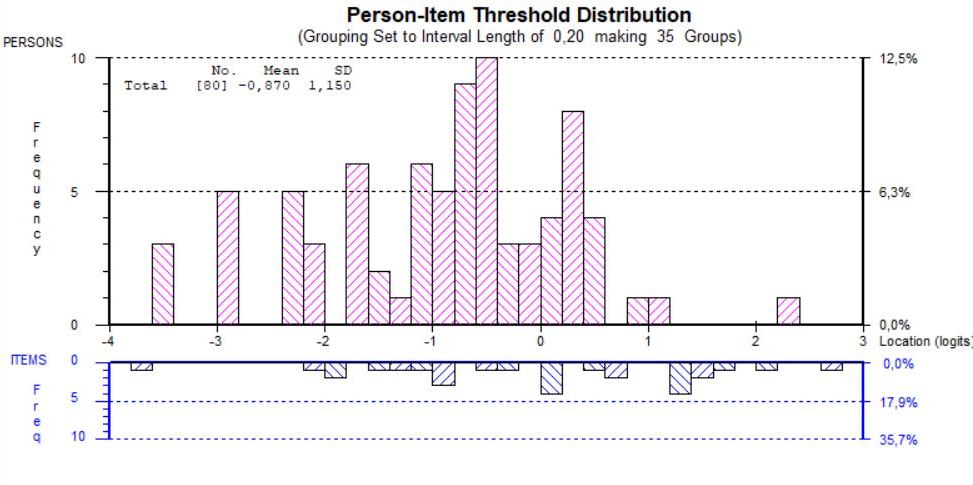
**

**Fig. 2** Person-Item Threshold Distribution of Rasch Analysis for ZBI-7

Supplement: Supplementary file 3 — (DOCX 86 kb) [file 520_2019_5288_MOESM3_ESM.docx]
